# Identification and management of inverted or everted edges of traumatic tympanic membrane perforations^☆^

**DOI:** 10.1016/j.bjorl.2017.10.002

**Published:** 2017-10-28

**Authors:** Zhengcai Lou, Zi-Han Lou

**Affiliations:** a The Affiliated YiWu Hospital of Wenzhou Medical University, Department of Otorhinolaryngology, Yiwu, China; b Xinxiang Medical University, Department of Clinical Medicine, Xinxiang City, China

**Keywords:** Traumatic, Tympanic membrane perforation, Edge approximation, Spontaneous healing, Traumática, Perfuração da membrana timpânica, Aproximação da borda, Cicatrização espontânea

## Abstract

**Introduction:**

Most of traumatic tympanic membrane perforations have inverted or everted edges, however, the effects of inverted and everted edges on the spontaneous healing of the eardrum remain controversial.

**Objective:**

We investigated the influence of inverted or everted edges on the spontaneous healing of traumatic tympanic membrane perforations.

**Methods:**

The clinical records of patients with a traumatic tympanic membrane perforations who met the study criteria were retrieved and categorized into two groups, based on whether the eardrum was inverted or everted. The features along the edge of each inverted or everted eardrum were described using 30° and 70° endoscopes.

**Results:**

In total, 196 patients (196 ears) met the inclusion criteria; of these, 148 had inverted or everted eardrums while 48 did not. Of the 148 patients with inverted or everted eardrums, the perforation edges were everted in 77 patients, inverted in 44 patients, drooping in 17 patients, and both inverted and everted in 10 patients. The perforation shape was triangular in 18.9% of patients, sector-shaped in 11.5%, kidney-shaped in 14.2%, ovoid in 20.3%, and irregularly shaped in 35.1% of patients. The difference was not significant between the with and without inverted/everted eardrum edges groups in terms of the closure rate or closure time. Similarly, the difference was not significant between the with and without edge approximation groups in terms of the closure rate or closure time at the end of the 12-month follow-up period.

**Conclusion:**

This study suggests that endoscopic inspection can clearly identify inverted/everted eardrum edges using 30° and 70° endoscopes. The edge is glossy in inverted/everted eardrums, whereas the edge is rough and irregular in non-inverted/everted cases. The inverted/everted eardrums gradually became necrotic, but this did not affect the healing process. Additionally, edge approximation did not improve the healing outcome of traumatic tympanic membrane perforations.

## Resumo

**Introdução:**

A maioria das perfurações de membrana timpânica traumáticas apresenta bordas invertidas ou evertidas; no entanto, os efeitos dessas configurações sobre a cicatrização espontânea do tímpano continuam a ser uma questão controversa.

**Objetivo:**

Investigar a influência de bordas invertidas ou evertidas sobre a cicatrização espontânea de perfurações traumáticas de membrana timpânica.

**Método:**

Os prontuários clínicos de pacientes com perfuração traumática de membrana timpânica que preencheram os critérios do estudo foram recuperados e categorizados em dois grupos, baseados na configuração invertida ou evertida das bordas da membrana timpânica. As características de configuração da borda de cada membrana foram descritas com o uso de endoscópios de 30° e 70°.

**Resultados:**

No total, 196 pacientes (196 orelhas) preencheram os critérios de inclusão; desses, 148 apresentavam bordas de membranas timpânicas invertidas ou evertidas, enquanto 48 não. Dos 148 pacientes, as bordas da perfuração estavam evertidas em 77 pacientes, invertidas em 44 pacientes, caídas em 17 pacientes e ambas invertidas e evertidas em 10 pacientes. O formato da perfuração era triangular em 18,9% dos pacientes, em forma de fatia de pizza em 11,5%, em forma de rim em 14,2%, ovoide em 20,3% e de forma irregular em 35,1% dos pacientes. A diferença não foi significante entre os grupos com e sem membrana timpânica invertida/evertida em termos de taxa ou tempo de fechamento. Da mesma forma, a diferença não foi significativa entre os grupos com e sem aproximação das bordas em termos de taxa de fechamento ou tempo de fechamento no fim do período de seguimento de 12 meses.

**Conclusões:**

Este estudo sugere que a avaliação com endoscópios de 30° e 70° pode identificar claramente as bordas invertidas/evertidas das perfurações de membranas timpânicas. A borda da perfuração timpânica em casos invertidos/evertidos é brilhante, enquanto a borda é áspera e irregular em casos não invertidos/evertidos. O rebordo timpânico invertido/evertido gradualmente torna-se necrótico, mas isso não afetou o processo de cicatrização. Além disso, a aproximação das bordas não melhorou o resultado da cicatrização.

## Introduction

The tympanic tembrane (TM) is extremely sensitive to pressure changes in the external auditory canal (EAC). Barotrauma may result in a TM Perforation (TMP) with inverted or everted edges.^1^, ^2^ Traumatic TMPs tend to heal spontaneously. However, the effects of inverted and everted edges remain controversial. Many surgeons believe that inverted or everted edges cause the centrifugal migration of epithelial cells, failure to close, and middle ear cholesteatoma.^3^, ^4^ Others have suggested that inverted and everted edges should be restored to the original anatomical position, thereby shortening the closure time and improving the closure rate.^5^, ^6^ However, the identification of inverted and everted edges is sometimes difficult. Additionally, the prognosis for inverted and everted edges is unclear. This study evaluated how to identify inverted and everted edges and the prognosis of inverted and everted edges in human traumatic TMPs.

## Methods

This study was reviewed and approved by the institutional ethical review board of XX Central Hospital (n° 20150608). Informed consent was obtained from all participants prior to enrolment.

### Case selection

Clinical records from traumatic TMP patients who presented to the Otolaryngology Outpatient Clinic at Hospital between August 2008 and August 2014 were accessed through the Records Department of the Hospital. Cases that met the following inclusion criteria were retrieved for analysis: (1) Traumatic TMP resulting from a slap or punch to the ear, or from a firecracker blast injury, within 7 days of the injury; (2) Spontaneous healing with or without edge approximation; (3) Recorded otoendoscopic video images of the healing process twice weekly until closure of the perforation or a 12 month follow-up period was available; and (4) The perforation size was graded as small, medium, or large (defined as <1/8, 1/8, −1/4, and >1/4 of the eardrum, respectively).^5^

### Data analysis

All clinical records and otoendoscopic videos of the patients were made available by the Records Department. The size and position of each perforation, the existence of inverted or everted edges, and the ultimate healing outcome were estimated by an independent, blinded reviewer using ImageJ software (AutoCAD R14). Each perforation was categorized as having or not having an inverted or everted edge based on the status of the edge from the remnant eardrum and perforation edge on the first visit and in the healing process, as seen on the video images. The perforation margin was categorized as an inverted edge (the edge is in contact with the mucosal layer), everted edge (the edge is in contact with the epithelial layer), or drooping edge (the edge is suspended in the tympanic cavity), based on the contact state of the edge. Additionally, the shape of the perforation was categorized as triangular, sector or kidney shaped, round, ovoid, or irregularly shaped, based on endoscopic observations. The connection length was divided into three types based on the correlation of the inverted or everted eardrum and the perforation margin: Type I (pedicle type), the connection length was <1/2 of the maximum diameter of the inverted or everted eardrum; Type II, the connection length was >1/2 of the maximum diameter of the inverted or everted eardrum; or Type III, the connection length was approximately equal to the maximum diameter of the inverted or everted eardrum.

The demographic characteristics of the patients, including age, sex, and injury duration, were analyzed as categorical data using the *χ*^2^ test. The primary outcome measures assessed were the extent of healing at the end of the 12 months follow-up period and the average healing time. These outcome data were analyzed using a two-sample comparison *t*-test. All tests were performed using SPSS software (SPSS Inc., Chicago, IL, USA). Differences were considered statistically significant at *p* < 0.05.

### Technical methods

The EAC was cleaned with a cotton bud soaked in povidone-iodine solution at the first visit. The remnant tympanic membrane and tympanic cavity were examined using 2.7 mm diameter, 0°, 30°, and 70° endoscopes; the edge of the perforation was subjected to gentle negative pressure using a microscopic suction tip to detect whether an inverted or everted eardrum was present at the perforation edge. Conservative treatment was adopted after endoscopic observation.

## Results

In total, 196 patients (81 males and 115 females) met the inclusion criteria. Their average age was 35.7 ± 5.8 (range 12–65) years. There were 183 dry perforations and 13 moist perforations with bloody otorrhea. The time from injury was within 1 day in 21 (10.7%) ears, 1–2 days in 45 (23.0%) ears, 3–4 days in 91 (46.4%) ears, and 5–7 days in 39 (19.90%) ears. Of the 196 perforations, the perforation size was small in 41 (20.9%) patients, medium in 103 (57.7%), and large in 52 (26.5%) patients. Demographic data for the groups are shown in Table 1. The following variables were similar between the two groups: age, sex, perforation size, and time since the injury (*p* > 0.05).

**Table 1:** Demographics of the patients in the with and without everted edge group.

Group	Without everted edge group	With everted edge group	*p*-value
N°	48	148	–
Sex, male/female	19/29	62/86	0.61^a^
Age, y	36.1 ± 3.8	35.9 ± 4.7	0.679^b^
Duration, d	3.8 ± 2.2	3.7 ± 2.9	0.55^b^
Perforated ear: left/right	32/16	127/21	0.71^a^
Perforation sized: S/M/L	17/26/5	41/86/21	0.52^a^

*p* < 0.05, was considered to indicate statistical significance.

a *χ*^2^ test.

b One-way analysis of variance.

Of the 196 ears, the perforation edge was glossy in 148 (75.51%) ears and rough and irregular in 48 (24.49%). An inverted epithelial layer or everted mucosal layer was found using a microscopic suction tip in the 148 patients with glossy edges, while neither an inverted nor an everted eardrum was seen in the 48 patients with rough and irregular edges.

**Figure 1:**
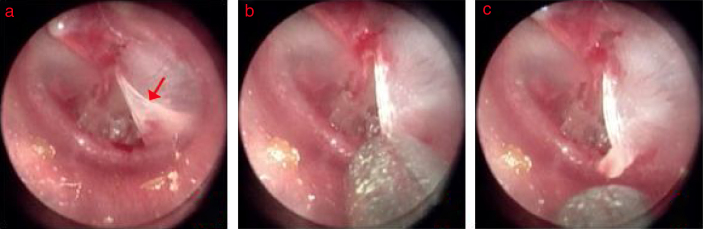
The process of edge approximation in the triangle perforation with everted eardrum: (a) 2nd days after perforation; (b) edge approximation; (c) everted eardrum was raised. Red arrows indicated everted eardrum.

**Figure 2:**
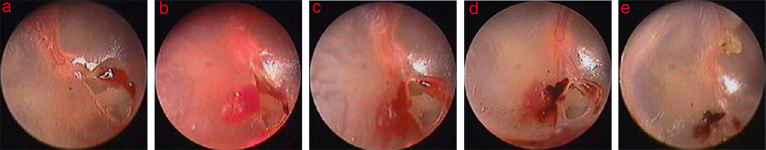
The healing process after edge approximation in the triangle perforation with everted eardrum: (a) 1st day after perforation; (b) edge approximation; (c–e) 1, 4, and 14 day after edge approximation.

**Figure 3:**
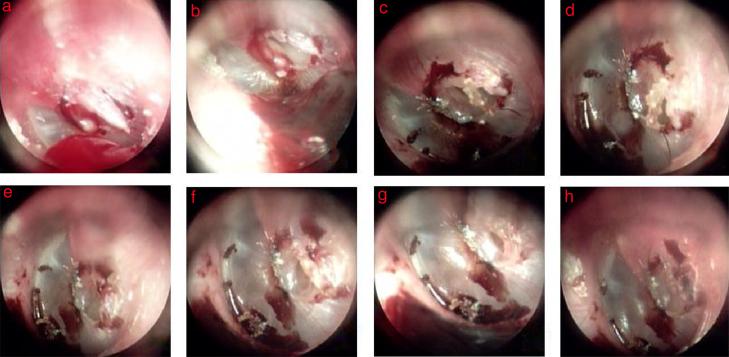
The spontaneous healing of spindle-shape perforation with drooping eardrum: (a–h) 3, 4, 6, 8, 10, 12, 14, and 16 days after perforation.

**Figure 4:**
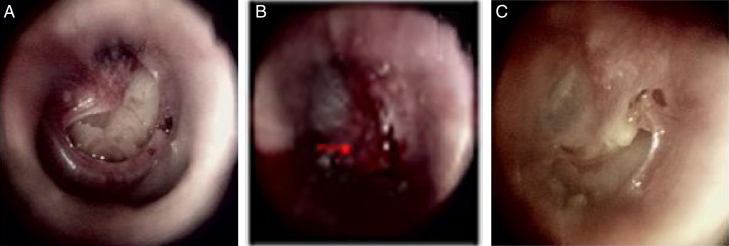
The healing process after edge approximation in the kidney-shaped perforation with inverted eardrum: (A) 1st day after perforation; (B) edge approximation; (C) 6 days after edge approximation. Red arrows indicated inverted eardrum.

Of the 148 patients with an inverted or everted eardrum edges, the perforation edge was everted in 77 patients (52.0%) (Figure 1, Figure 2), drooping in 17 (11.5%) (Fig. 3), inverted in 44 (29.7%) (Fig. 4), and both inverted and everted in 10 (6.8%) patients. In the 148 patients with an inverted or everted eardrum, the membrane was restored to its original anatomical position within 3 days after injury in 63 (42.6%) patients but not in 85 (57.4%) patients. The perforation shape was triangular in 28 (18.9%) patients, sector-shaped in 17 (11.5%), kidney shaped in 21 (14.2%), ovoid in 30 (20.3%), and irregularly shaped in 52 (35.1%) patients. In the 48 patients without an inverted or everted eardrum, the perforation shape was ovoid in 11 (22.9%), round in 9 (18.8%), and irregularly shaped in 28 (58.3%) patients. The size of inverted or everted eardrum was >∼80% of the size of the perforation in 21 (14.2%) patients, 50–80% in 59 (39.9%), 20–50% in 52 patients (35.1%), and <20% in 16 (10.8%). The inverted or everted eardrum turned in the direction of the pars tensa or malleus in 107 (72.3%) patients, toward to the tympanic annulus in 24 (16.2%), and toward the tympanic cavity in 17 (11.5%). The connection length was Type I in 11 (7.4%) patients, Type II in 38 (25.7%), and Type III in 99 (66.9%).

### Healing outcome

Patients were followed for a total of 12 months or until complete closure of the perforation. The healing outcome is shown in Table 2, Table 3. The closure rate at the end of the 12 months follow-up period was 93.8% for perforations without an inverted or everted eardrum and 92.6% for perforations with an inverted or everted eardrum; the difference was not significant (*p* > 0.05). The average closure time was not significantly different (29.4 ± 3.7 vs. 31.2 ± 5.8 days, *p* > 0.05) between the with and without inverted or everted eardrum groups. Additionally, in terms of the healing outcome of the 148 patients with and without edge approximation, the closure rate was not significantly different (94.1% vs. 90.5%, *p* > 0.05), nor was the closure time (30.6 ± 5.7 vs. 32.1 ± 4.2 days, *p* > 0.05).

**Table 2:** The healing outcome of without and with everted edge group.

	Without everted edge group	With everted edge group	*p*-value
N°	48	148	–
Closure rate,%	45 (93.8)	137 (92.6)	0.35^a^
Closure time, days	31.2 ± 5.8	29.4 ± 3.7	0.216^b^

*p* < 0.05, was considered statistically significant.

a *χ*^2^ test.

b *t*-test.

**Table 3:** The healing outcome of with and without edge approximation group.

	With edge approximation group	Without edge approximation group	*p*-value
N°	63	85	–
Closure rate,%	80 (94.1)	57 (90.5)	0.81^a^
Closure time, days	30.6 ± 5.7	32.1 ± 4.2	0.47^b^

*p* < 0.05, was considered statistically significant.

a *χ*^2^ test.

b *t*-test.

### Endoscopic observations

For 85 patients without edge approximation, the inverted or everted eardrum became black during the spontaneous healing process within 3 days. Thereafter, the black everted eardrum gradually increased, and the inverted or everted eardrum became completely black and formed a crust in the Type I cases on days 4–5. The inverted or everted eardrum formed a complete crust in Type II and Type III cases on days 5–7. Proliferation and migration of the epithelium occurred simultaneously and increased gradually over time. Additionally, in the drooping eardrum cases, there was gradual shrinkage along the perforation edge and a crust formed over time (Fig. 3). The crust migrated gradually toward the EAC.

In the 63 patients with edge approximation, the perforation size reduced to ∼20–50% in 41 patients and >50% in 22 patients after edge approximation. The repaired eardrum had not obviously changed on day 2; the morphology of the inverted or everted edge was similar to that prior to approximation, but swelling occurred. The contracture of the repaired inverted or everted eardrum started to occur on day 3 in 56 patients. Proliferation and migration of the epithelium occurred simultaneously on the side with no inverted or everted edge, and contracture became increasingly obvious along the perforation edge over time, especially on an inverted or everted eardrum on the side of the tympanic annulus (Fig. 4). The repaired inverted or everted eardrum returned completely to the original edge and formed a crust about 1 week after approximation. Proliferation and migration of the epithelium then began. Proliferation and migration of the epithelium increased gradually on the side with no inverted or everted edge. The repaired inverted or everted eardrum did not shrink on the side of the annulus in seven patients. No proliferation or migration of the epithelium was seen in repaired inverted or everted eardrums during the healing process, and a strip-shaped perforation resulted in the seven patients.

## Discussion

A sudden, rapid change in pressure in the EAC may cause a TMP with an inverted or everted eardrum at the edges.^7^, ^8^ Researchers believe that the timely repair of an inverted or everted eardrum can reduce the size of the perforation, shorten the closure time, and improve the closure rate.^5^, ^6^ Thus, it is important to identify an inverted or everted eardrum at the edge. With the development of endoscopic examination, identifying an inverted or everted eardrum is now possible. Endoscopic examination, using 30° and 70°endoscopes, can detect inverted eardrums and ossicular chain interruption in the tympanic cavity through the perforation. We found that the edges of the perforations had the following characteristics: (1) The edge was glossy in inverted or everted eardrums, whereas the edge was rough and irregular in non-inverted or everted cases; and (2) Perforations with a triangular, sector shape, or kidney shape were typically inverted or everted eardrum cases, whereas a round perforation was common in non-inverted or everted eardrum cases. In addition, inverted or everted eardrums could typically be identified with the help of suction using a microscopic suction tip.

The management of inverted or everted eardrums edges is controversial. Many researchers^3^, ^4^ believe that this can affect the spontaneous healing of a TMP and the development of middle ear cholesteatoma. Others have suggested that an inverted or everted eardrum does not affect the healing outcome of TMPs.^9^ Thus, most scholars have advocated that an inverted or everted eardrum should be restored to its original anatomical position to reduce the size of the perforation. However, this study shows that an inverted or everted eardrum did not affect spontaneous healing. The difference was not significant between the with and without inverted or everted eardrum groups in terms of the closure rate and closure time. Similarly, the difference was not significant between the with and without edge approximation groups in terms of the closure rate and closure time. We found that the inverted or everted eardrum gradually became black, with a crust forming over time during the healing process. Proliferation and migration of the epithelium occurred and increased gradually on the side with no inverted or everted eardrum; proliferation and migration of the epithelium also began on the side of the inverted or everted eardrum until a complete crust was formed. The prognosis may be related to the blood supply of the inverted or everted eardrum. The blood vessels of the eardrum are distributed around the malleus and annulus; the pars tensa has few vessels.^10^, ^11^ The blood supply to an inverted or everted eardrum is limited after a TMP; the inverted and everted eardrums in this study gradually became necrotic and formed a crust, resulting from hypoxia-ischemia. The time of necrosis was lengthened in Type III inverted or everted eardrums, whereas Type I inverted or everted eardrums rarely became completely necrotic within 3 days. Additionally, necrosis in the eardrum did not affect spontaneous healing, but it migrated toward the EAC. Regarding whether an inverted or everted eardrum affected spontaneous healing in a wet middle ear with secondary bloody otorrhea, the wet environment maintained a microenvironmental balance, avoiding necrosis at the edge.^12^, ^13^ However, we found that everted eardrums liquefied gradually and disappeared with moist or wet perforations over time; this did not seem to affect spontaneous healing. However, it was difficult to assess whether an inverted or drooping eardrum could grow into the tympanic cavity in a wet middle ear because there were few cases of wet perforations and shorter follow-up times. Nevertheless, some researchers^14^, ^15^ have reported that a wet environment in the middle ear increases the likelihood of middle ear cholesteatoma. Kronenberg^4^ concluded that the risk of cholesteatoma development was highest with kidney-shaped perforations. We found that kidney-shaped perforations were always accompanied by inverted or drooping eardrums; it is possible that an inverted or drooping eardrum grew into the tympanic cavity in a wet middle ear.

Regarding whether edge approximation can accelerate eardrum healing, we found that although edge approximation reduced the size of the perforation in the early stages, the repairing inverted or everted eardrum shrank gradually along the edge and retracted completely to the edge and formed a crust after 1-week, and the perforation size increased again. Surprisingly, proliferation and migration of the epithelium on the side of the edge approximation was slower than that on the side with no inverted or everted eardrum. Another situation we encountered was that although retraction of the repairing inverted or everted eardrum did not occur, proliferation and migration of the epithelium did not begin on the side to repair the inverted or everted eardrum during the healing process; perforation healing depended on proliferation and migration of the epithelium from the other side of the non-repaired inverted or everted eardrum. As a result, strip-shaped perforations remained. Recently, several clinical studies have demonstrated that edge approximation did not improve the closure rate of traumatic TMPs.^4^, ^16^, ^17^ Thus, edge approximation apparently has little significance in the healing of traumatic TMPs in a clinical setting.

This study has some limitations. First, the sample numbers were not balanced. Second, the assessment and comparison of healing outcomes among the groups were based on all of the perforations in each group, but did not consider perforation size.

## Conclusion

Our findings suggest that endoscopic inspection can clearly identify inverted or everted eardrums using 30° and 70° endoscopes. The edge was glossy in inverted or everted eardrums, whereas the edge was rough and irregular in non-inverted or everted eardrum cases. Those perforations with a triangular, sector shape, or kidney-like shape exhibited an inverted or everted eardrum. In addition, an inverted or everted eardrum was usually identified with the help of suction using a microscopic suction tip. Each inverted or everted eardrum gradually became necrotic and formed a crust over time. This did not affect the healing process. Additionally, the repairing inverted or everted eardrum shrank continually over time and retracted to the edges. Proliferation and migration of the epithelium did not occur or was delayed on the side of edge approximation, but edge approximation did not improve the healing outcome of traumatic TMPs.

## Funding

This study was supported by the Science and Technology Agency of Yiwu City, China (Grants n° 15-3-306).

## Conflicts of interest

The authors declare no conflicts of interest.
